# Biobanking in the emergency department: implementation of the Mayo Clinic Emergency Department Sepsis Biorepository

**DOI:** 10.1186/cc14044

**Published:** 2014-12-03

**Authors:** CM Clements, JR Anderson, J Uhl, MI Rudis, FR Cockerill

**Affiliations:** 1 Department of Emergency Medicine, Mayo Clinic, Rochester, MN, USA

## Introduction

Biomarker discovery research has not focused on the emergency department (ED) due to perceived lack of access to ED patients for study, difficult patient identification, and preemption by time-critical clinical needs. The aim of this study was to use an automated process to accrue, within the ED, a biobank of patients presenting at risk for severe sepsis.

## Methods

Our group has previously derived an algorithm for identification of at-risk patients of which about 45% will be severely septic [1]. Here, we utilize that algorithm to identify a prospective cohort to be included in our ED sepsis biobank. Patients are excluded if they are pregnant, <18 years old, a vulnerable adult, or imprisoned. An electronic notification system identifies patients and automatically pages phlebotomy and laboratory processing personnel directly. Blood draws occur immediately after patient identification and at 6 and 24 hours into hospitalization. We use an IRB-approved delayed consent model that minimizes the time between identification and blood draw. Plasma extracts and cellular isolates are processed and stored immediately. Clinical data are extracted for relevant patient outcomes, severe sepsis, and indicators of critical illness.

## Results

In 1 year of accrual, the alert algorithm identified 1,773 eligible patients for screening. Blood was drawn from 873 patients based on phlebotomy availability and laboratory processing staffing. A total of 642 patients completed blood draws and consented for inclusion, with the balance of patients having been discharged, transferred, or deceased with no identifiable legally authorized representative for consent. The median time from alert to first blood draw was 25 minutes (range 5 to 60 minutes). In an observation period, during which 227 consecutive patients had blood drawn, only 12 patients or families (5.2%) declined consent after the initial blood draw.

## Conclusion

We have successfully developed and implemented a biobank in the ED that functions within practice limitations and has high patient accrual. Automation is key for efficient implementation in the ED. Using automated design improvements, we obtain blood samples from sepsis patients presenting to the ED faster than previous studies. Prompt biobank collection within the ED allows research into mechanisms of sepsis earlier in a patient's hospital course than has heretofore been possible and enables biomarker discovery for testing development directly relevant to emergency providers.
